# Systematic Review of the Effect of a Zero-Markup Policy for Essential Drugs on Healthcare Costs and Utilization in China, 2015–2021

**DOI:** 10.3389/fmed.2021.618046

**Published:** 2021-07-22

**Authors:** Wen-Yi Liu, Chia-Hsien Hsu, Ting-Jun Liu, Pei-En Chen, Boyuan Zheng, Ching-Wen Chien, Tao-Hsin Tung

**Affiliations:** ^1^Department of Health Policy Management, Bloomberg School of Public Health, Johns Hopkins University, Baltimore, MD, United States; ^2^Institute for Hospital Management, Tsing Hua University, Shenzhen, China; ^3^Shanghai Bluecross Medical Science Institute, Shanghai, China; ^4^Department of Public Health, College of Health Science, Kaohsiung Medical University, Kaohsiung, China; ^5^Tai Kang Institute of Healthcare Management, Beijing, China; ^6^Institute of Health Policy and Management, College of Public Health, National Taiwan University, Taipei, Taiwan; ^7^Evidence-Based Medicine Center, Taizhou Hospital of Zhejiang Province Affiliated to Wenzhou Medical University, Linhai, China

**Keywords:** zero-markup policy, medical expenditure, systematic review, China, healthcare

## Abstract

**Objective:** This systematic review aimed to discuss the effects of a zero-markup policy for essential drugs (ZPED) on healthcare costs and utilization in China in the years 2015–2021.

**Methods:** We searched the PubMed, Embase, Scopus, and CINAHL databases for all associated studies carried out from January 1, 2015, to May 31, 2021, without any limitations regarding the language the studies were written in. To prevent selection bias, gray documents were tackled by other means. The methodological approaches were assessed by applying the Preferred Reporting Items for Systematic Reviews and Meta-Analyses (PRISMA) guidelines and the Newcastle-Ottawa Scale (NOS) collaboration tool.

**Results:** Forty studies were selected at first and then 15 studies that met the inclusion criterion. Most of the studies showed a considerable decrease in total medical spending and drug spending in both outpatient and inpatient services. After the implementation of ZPED, studies showed that the medical services increased and total hospital income sustained, despite a decrease in drug revenue. Minimal or no government subsidy is required from a financial perspective.

**Conclusions:** Although, the government could implement ZEPD with lower medical cost and drug cost to patients, and sustained income for health facilities, we have limited understanding of whether the increase in medical services was induced by the provider or was a response to unmet needs in the population. Further, studies using rigorous and advanced methods to study health policy, patient behaviors, provider behaviors, and government decisions are warranted.

## Introduction

The Policy on Drug Markups (PoDM) implemented by the government in 1954 allowed medical institutions in China to increase drug prices by a maximum of 15% (1). From the 1980's, it became evident that patients were struggling with obtaining medical care due to the insufficient fiscal allowance and increases in drug costs (2). The Chinese government launched a campaign of clinical health modernization to mitigate these problems and implemented the zero-markup policy for essential drugs (ZPED) in 2009. The ZPED mandated that the markup from medication bills could no longer be retained and that 10% of the original 15% markup under the PoDM would be substituted by fiscal allowance. The answer for the remainder of the discharge was believed to be a preposterous method, and it was thought that diagnostic expenses would be increased to obtain 80% of the former markup. The others were the main parts of the hospitals themselves (3).

Even though the implementation of ZPED was essential to restrict the increase in drug prices, it could not prevent patients from experiencing financial difficulties, which are now caused by paying for other treatments rather than expensive drugs. For instance, when the policy was initially implemented (2009–2011), the pharmaceutical fees of patients per visit were decreased, particularly in rural areas and counties. An evidence-based study showed that the substitution effect of medical consumables offsets the decrease in total expenditure in the long term (4). Hospitals that rely on earnings by drugs to cover their costs could be mitigated by ZPED (5). From 2011 to 2015, this strategy was no longer a pilot scheme and came to be used in all county-level hospitals in China. However, medical expenses were still rising, in spite of efforts to control the increase (2). This meant that hospitals were able to increase their spending on other medical areas covered by ZPED, such as discharge diagnosis fees, nursing payments, surgery expenses, and treatment fees (2).

Most previous studies on this topic focused on the enduring effects of ZPED on expenses of patients per visit, in particular, drug costs, but neglected the overall cost of the process of therapy. Therefore, this systematic review aimed to discuss the effects of ZPED on healthcare costs and utilization in China from 2015 to 2021.

## Materials and Methods

### Data Sources and Selection

We searched the PubMed, Embase, Scopus, and CINAHL databases for all associated studies carried out from January 1, 2015, to May 31, 2021, without any limitations regarding the language the studies were written in. To prevent selection bias, gray documents—for example, OpenGrey and Open Access Theses and Dissertations—were tackled by other means. We performed a search of the aforementioned electronic databases, applying keywords included in the title and/or abstract as follows: (“pharmaceutical^*^” OR “drug^*^” OR “medicine^*^”) AND [(“zero”) AND (“markup” OR “mark-up”) AND (“China”)]. The selection sheets of the associated studies were evaluated manually to identify comparable works (Table 1). Due to the fact that no study patients were enrolled, as we only used published studies, it was not necessary for us to obtain approval from the institutional review board (IRB) for this systematic review. Two reviewers evaluated regular studies that assessed the effects of ZEPD on the annual medical expenditure per subject and the expense of courses of therapy. Disagreements were resolved *via* conversation with a well-trained third reviewer. The studies we selected were original articles rather than letters to the editor, editorials, commentaries, or congress documents. The results of these included investigations should be related to the financial indicators of medical institutions. For instance, we aimed to find data on the total expense per inpatient or outpatient visit, the costs of drugs per visit, the number of visits a patient required, etc.,

**Table 1:** Search strategy in PubMed up until 31th May 2021 (similar search run in other databases).

1 “Pharmaceutical*” [Title/Abstract]
2 “Drug*” [Title/Abstract]
3 “Medicine*” [Title/Abstract]
4 1 OR 2 OR 3
5 “Zero” [Title/Abstract]
6 “Markup” [Title/Abstract]
7 “Mark-up” [Title/Abstract]
8 6 OR 7
9 “China” [Title/Abstract]
10 4 AND 5 AND 8 AND 9

### Data Extraction and Quality Assessment

The Newcastle-Ottawa Scale (NOS) was used for quality assessment (6). This is an approach that has been proven to be effective for appraising methodological quality in non-randomized controlled trials. The two reviewers also summarized the relevant features of the selected studies using a standardized data collection form. Table 2 indicates the results of the quality ratings. Each asterisk means one star, and the total scale of NOS is the summation of the stars (nine is the maximum), which are allocated for selection (four stars), comparability (two stars), and outcome (three stars).

**Table 2:** Quality assessment of included studies using the Newcastle-Ottawa Scale (NOS).

**Source**	**Selection**				**Comparability**	**Exposure**			**Total NOS score**
	**(1)**	**(2)**	**(3)**	**(4)**	**(1)**	**(1)**	**(2)**	**(3)**	
Zhou et al. 2015 (5)	⋆	⋆	⋆	⋆	⋆⋆	⋆	⋆		8
Zhou et al. 2015 (7)	⋆	⋆	⋆	⋆	⋆⋆	⋆	⋆		8
Tian et al. 2016 (8)			⋆			⋆			2
Wei et al. 2017 (9)	⋆	⋆	⋆		⋆⋆	⋆			6
Yang et al. 2017 (2)	⋆		⋆			⋆			3
Fu et al. 2018 (10)	⋆		⋆		⋆⋆	⋆			5
He et al. 2018 (3)	⋆		⋆			⋆			3
Tang et al. 2018 (1)			⋆			⋆			2
Yin et al. 2018 (11)	⋆		⋆			⋆			3
Mao et al. 2019 (12)	⋆		⋆			⋆			3
Shi et al. 2019 (13)	⋆		⋆		⋆⋆	⋆			5
Zeng et al. 2019 (4)	⋆	⋆	⋆	⋆	⋆	⋆			6
Jiang et al. 2020 (14)	⋆		⋆		⋆⋆	⋆	⋆		6
Li et al., 2021 (15)	⋆		⋆		⋆⋆	⋆			5
Du et al., 2021 (16)	⋆		⋆		⋆⋆	⋆			5

***Selection***

*(1) Representativeness of the exposed cohort*.

*(2) Selection of the non-exposed cohort*.

*(3) Ascertainment of exposure*.

*(4) Demonstration that outcome of interest was not present at start of study*.

***Comparability***

*(1) Comparability of cohorts on the basis of the design or analysis*.

***Exposure***

*(1) Assessment of outcome*.

*(2) Was follow-up long enough for outcomes to occur*.

*(3) Non-response rate*.

### Data Synthesis

Four extensive outcomes were considered: (1) medical cost; (2) drug cost; (3) healthcare utilization; and (4) others (facility revenue, drug revenue, and government subsidy). The baseline and intervention for the outcome variables were evaluated.

## Results

### Characteristics of the Included Studies

**Figure 1:**
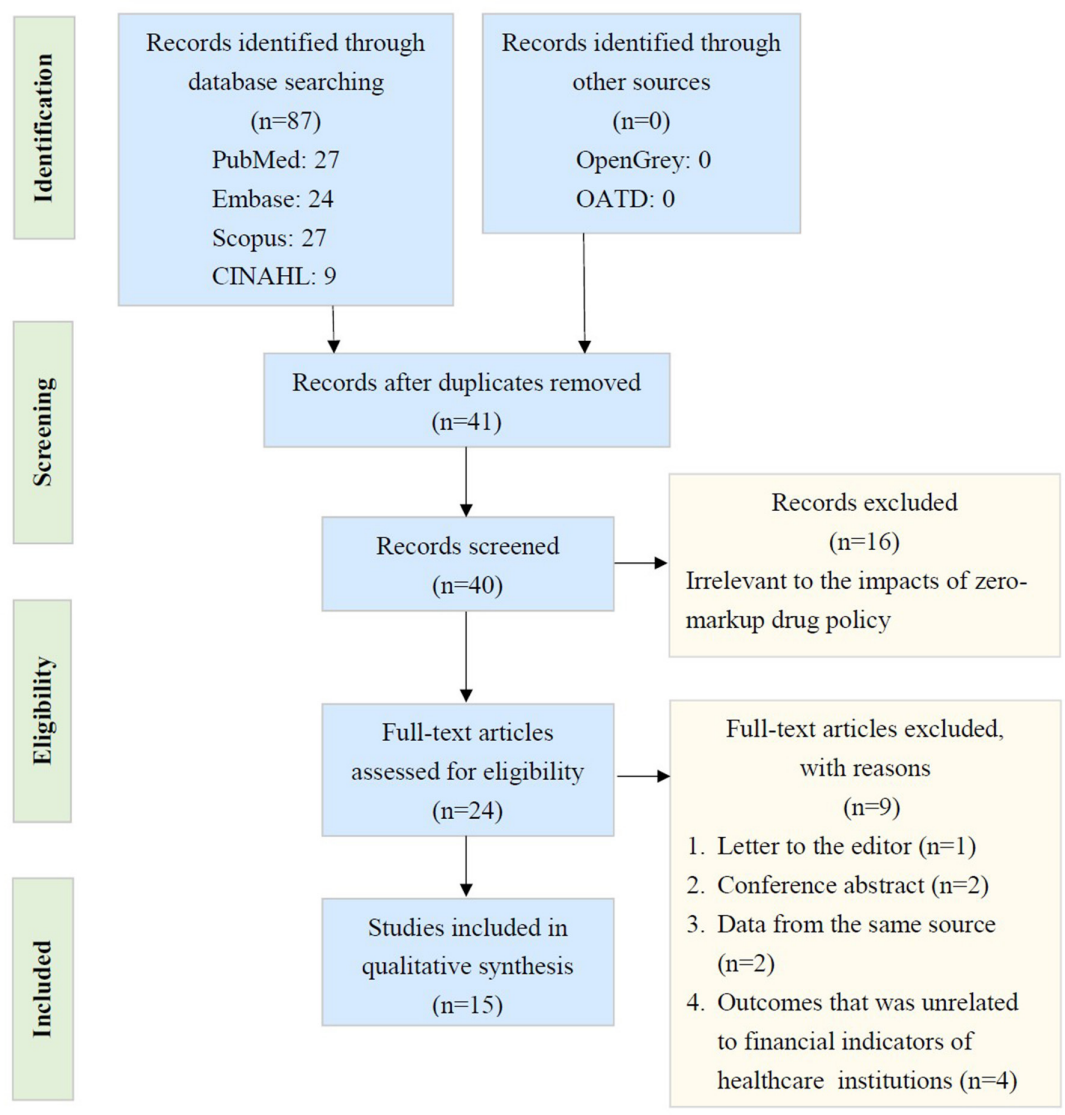
PRISMA (Preferred Reporting Items for Systematic Review and Meta-Analyses) flow diagram. (CINAHL: Cumulative Index to Nursing and Allied Health Literature; QATD: Open Access Theses and Dissertations).

As shown in Figure 1, our investigation started with 40 records after ruling out repeats. We discarded 25 records that could not fulfill the selection criteria. Fifteen studies (including retrospective cohort studies, time series studies, and quasi-experimental studies) were eventually included (1–5, 7–16). Table 3 outlines the features of the selected studies. All of the studies included were from China. Not all studies were classified as having more than seven stars based on the NOSs, and some were believed to be of lower quality. Table 3 also shows the various estimated results.

**Table 3:** Characteristics of included studies in China.

**Author**	**Publication year**	**Study design**	**Area**	**Hospital accreditation**	**Outcomes**	**Statistical methods**	**Conclusions**
Zhou et al. (5)	2015	Quasi-experimental study	Ningshan County, Zhenping County, and Shaanxi Province	Secondary	• Total expense per visit (inpatient/outpatient service). • Drug expense per visit (inpatient/outpatient service).	• Hospital-data difference-in-differences. • Individual-data regressions.	• The absolute monetary reduction of the per-visit inpatient expense is 20 times of that in outpatient care. • The relative reductions are the same for outpatient and inpatient visits. • The incentive to utilize outpatient or inpatient care attributed to Zero-markup Policy for Essential Drugs is equivalent, regardless of the price difference in absolute terms.
Zhou et al. (7)	2015	Quasi-experimental study	Ningshan County Hospital in Ankang city, Shaanxi province		• The effects of zero-markup on medical expense per visit. • The effects of zero-markup policy on medical service provision. • The effects of zero-markup policy on the health care revenue for hospitals. • Estimates of government subsidy.	• A difference-in-difference model to measure the difference in several indicators between two hospitals.	With minimal or no subsidy, the government can catalyze the zero-markup policy and potentially generate positive outcomes for county hospitals.
Tian et al. (8)	2016	Descriptive study (retrospective longitudinal study)	Beijing	Tertiary	• Annual patient-visits. • Ratios of medicine-to-healthcare-charges (RMOH). • Physician work-days (inpatient/outpatient service). • Physician-workload (inpatient/outpatient service). • Inflation-adjusted per-visit healthcare charges (inpatient/outpatient service). • Mortality-rate (inpatient/outpatient service).	• Rank-sum tests. • Join-point regression analyses.	Implementation of Universal Zero-Markup Drug Policy: • Increase annual patient-visits • Reduce RMOH • Have different impacts on outpatient and inpatient services
Wei et al. (9)	2017	Natural experiment	Guangxi	N/A	• Antibiotic prescribing rate (outpatients with a primary diagnosis of upper respiratory tract infection).	• Difference-in-difference analyses.	The national essential medicines scheme and zero-mark-up policy may be associated with reductions in outpatient antibiotic prescribing and intravenous infusions.
Yang et al. (2)	2017	Time series study	Shaanxi Province	Primary	• Monthly average hospitalization expenditure (AHE). • Monthly average hospitalization expenditure after reimbursement (AHER).	• Segmented regression analysis of interrupted time series data.	A statistically significant absolute decrease in the level or trend of monthly AHE and AHER was detected after the introduction of the zero-markup drug policy in western China. However, hospitalization expenditure and hospitalization expenditure after reimbursement were still increasing. More effective policies are needed to prevent these costs from continuing to rise.
Fu et al. (10)	2018	Penal study	1,880 counties	N/A	**Outpatient care** • Total expenditures per visit. • Drug expenditures per visit. • Expenditures for diagnostic tests/medical consumables per visit. • Expenditures for medical services per visit. • Outpatient visits. **Inpatient care** • Total expenditures per admission. • Drug expenditures per admission. • Expenditures for diagnostic tests/medical consumables per admission. • Expenditures for medical services per admission. • Inpatient admissions. • Average length of inpatient stay.	• Pre-trend test based on linear regressions.	The policy change led to a reduction in drug expenditures, a rise in expenditures for medical services, and no measurable changes in total health expenditures. However, this study also found an increase in expenditures for diagnostic tests/medical consumables at hospitals that had a greater reliance on drug revenues before the reform, which is unintended by policymakers. Further, these results were more likely to be driven by the supply side, suggesting that hospitals offset the reductions in drug revenues by increasing the provision of services and products with higher price-cost margins.
He et al. (3)	2018	Time series study	Sanming City, Fujian Province	Secondary (*n* = 4) and tertiary (*n* = 21)	• Outpatient drug expenditure. • Outpatient total health expenditure. • Inpatient drug expenditure. • Inpatient total health expenditure.	• Interrupted time series analysis with three segments divided by two intervention points.	Although, the pharmaceutical reform could control or reduced drug expenditure and total health expenditure in short term, expenditures gradually resumed growing again and reached or even exceeded their baseline levels of pre-reform period, indicating the effect became weakened or even faded out in long term.
Tang et al. (1)	2018	N/A	Nanjing City, Jiangsu Province	Secondary and tertiary	• The markup ratio of drug sales. • The growth rate of medical service revenue.	• Simple linear interrupted time series regressions.	Nanjing's pricing and compensation reform has basically achieved the policy targets of eliminating the drug markups, promoting the growth of medical services revenue, and adjusting the structure of medical revenue. However, the growth rate of service revenue of hospitals varied significantly from one another.
Yin et al. (11)	2018	Time series study	Shandong Province	Secondary tertiary, and urban/rural primary healthcare centers (PHCs)	• Total annual antibiotic expenditure. • Antibiotic expenditure per person per year.	• Descriptive statistics.	The overall antibiotic expenditure increased over time in Shandong, China. However, the increase rate of expenditure began to decline in 2016, possibly related to the implementation of antibiotic stewardship initiatives.
Mao et al. (12)	2019	Penal study	Hangzhou City, Zhejiang Province	Primary (*n* = 6), secondary (*n* = 2), and tertiary (*n* = 9)	• Average number of medicines. • Average number of antibiotics. • Average expenditure per prescription.	• *T*-test. • Pearson Chi-square test or Fisher exact test.	The average number of medicines per prescription, use of antibiotics, intramuscular (IM) injections and intravenous (IV) injections decreased while the use of hormones increased. No significant change of the average medicine expenditure per prescription was observed. The problems of poly-pharmacy, overuse of antibiotics, intramuscular (IM) injections and intravenous (IV) injections and hormones still existed, however mitigated after the implementation of The National Essential Medicine Policy and the Zero Mark-up Policy.
Shi et al. (13)	2019	Penal study	All TCM county hospitals		• Revenue from government subsidies. • Share of revenue from drug sales (divide the amount of revenue from TCM and Western medicine by total revenue except government subsidies). • Revenue from Chinese medicine sales. • Revenue from western medicine sales. • Revenue from medical care services. • Gross revenue. • The number of annual outpatient visits and the number of inpatient admissions.	• Difference-in-difference.	• ZMDP achieved its stated goal through reducing the share of revenue from drug sales without disrupting the availability of healthcare services at TCM county hospitals no matter in the short term or long term. • The success of ZMDP was mainly due to the huge growth in the government's financial investment in TCM hospitals, which offset western medicine sales revenue, while maintaining current hospital service levels. However, whether government financial investment can continue such long-term growth remains an open question.
Zeng et al. (4)	2019		Beijing City	Urban employee basic medical insurance	• The total expenditure and other expenditure components of the pilot hospitals.	• 1:1 propensity score-matched analysis (Propensity scores were calculated by logistic regression).	After the zero markup drug policy, expenditure on drugs revealed a continuous decline. However, the decline in total expenditure was weakened by the substitution effect of medical consumables in the long term.
Jiang et al. (14)	2020	Penal study	Shandong Province	Secondary and tertiary	• Revenue from medicine sales. • The share of revenue from medicine sales. • Evenue from medical care services. • Government subsidies. • Revenue and expenditure surplus. • Gross revenue. • The number of annual outpatient and inpatient visits.	• Difference-in-difference analyses.	• The ZMDP achieved its some initial goals of removing the profits from western medicines in county hospitals' revenue without disrupting the normal operation, and had different impacts between county general andTCM hospitals. • Meanwhile, some unintended consequences were also recognized through the analysis, such as the decline of the utilization of the TCM.
Li et al. (15)	2021	Penal study	Chengdu City	Urban employee basic medical insurance	• A series of expenditure variables (actual reimbursement expenditure, reimbursement ratio, total healthcare expenditures, drug expenditure, examinations expenditure, material expenditure, nursing expenditure, etc.,).	• Difference-in-difference analyses.	• After implementing the SHRDS policy, the significant reduction in drug expenditure led to more physicians inducing patients' healthcare service needs, and the increased social healthcare burden was partially transferred to the patients' personal economic burden through the decline in the reimbursement ratio. • The SHRDS policy is not an effective way to control healthcare expenditure.
Du et al. (16)	2021	Interrupted time series study	Chongqing City	Tertiary	• Average drug cost 11 per month for NCDs' outpatients analyzed overall.	• Interrupted time series analysis.	• The ITS analysis is an effective method of health policy evaluation. • The implementation of the ZMDP can reduce the drug cost for chronic disease outpatients in the tertiary hospital and their economic burden. • Follow-up policies still require targeted price adjustments in the health service system to adjust the drug cost effectively.

### Medical Cost

The total expenses decreased by 3.12 and 65.6 U.S. dollars for outpatients and inpatients, respectively, per visit according to the quasi-experimental study design. The expense of each visit was predicted to show a decrease of 11% for both outpatient and inpatient medical services (5). One retrospective follow-up study showed that there was an increase in the annual number of patient visits (8). Another study revealed that there was a significant reduction in the expense of hospitalization per month (2). In addition, an increase in expenditure on clinical services was noted, while no alteration in overall healthcare costs was observed (10). The medication reform did not manage to longitudinally decrease the overall health expenditure of the patients (3). The great growth in the investment of the government in hospitals offsets the revenue from the sales of Western medicine, meaning that the zero-markup drug policy (ZMDP) can be considered a success; however, whether the investment of the government can last for a long duration is a factor that needs to be considered (13). A positive relationship was also found between the compensation for the situation of services and the contents of the annual income of clinical services (1). However, no meaningful modification of the average medical expenditure per treatment was noticed (12).

### Drug Cost

One selected study revealed that not only the costs of drugs per patient visit declined by 4.47 U.S. dollars (outpatient services) or 45.75 U.S. dollars (inpatient services) but also the proportion of money spent on drugs out of the overall medical expenditure per patient visit decreased by 11.73% for outpatient visits and 3.92% for inpatient visits (5). Another study showed that the implementation of ZPED was associated with a reduction in the use of antibiotics (11). ZPED succeeded in removing the profits from Western medicines from the revenues of county hospitals without significantly disrupting their normal operation (17). In addition, the change in policy caused a reduction in drug expenditure (10). The medication reform was only able to decrease drug expenses in the short term (3). One study showed that the number of medicines prescribed per patient and the use of antibiotics, intramuscular injections, and intravenous injections reduced, while the use of hormones increased (12). The total drug expenditure decreased by 14.4%, while the drug expenditure of outpatients with chronic diseases in tertiary hospitals was reduced due to the implementation of the ZPED (14, 16).

### Healthcare Utilization

With regard to the reduction in the ratio of medicine to healthcare charges in services to patients, both outpatient and inpatient services showed increases in the annual number of patient visits (8). ZPED may be associated with decreases in antibiotic prescriptions and intravenous infusions for outpatient visits (9).

The provision of outpatient services increased in the treatment group by 28.55%, while the provision of inpatient services increased by 16.17%. The provision of outpatient services was similar to that of the control group, and the provision of inpatient services only increased by 1.31%. Following the implementation of a zero-markup policy, the provision of outpatient and inpatient services in the treatment group increased by 9,697 and 398 visits, respectively [27].

### Others

The great reduction in expenditure on drugs caused more physicians to induce patients/healthcare service needs. The separation of hospital revenue from drug sales (SHRDS) policy is not an effective means of controlling healthcare expenditure (16). In addition, the increase in inpatient physician workdays decreased the mortality rate of inpatients. Workloads and inflation-adjusted per visit medical care charges of physicians increased in the outpatient services (8).

## Discussion

### Clinical Implications of a Zero-Markup Policy

Few systematic reviews have been conducted to explore the effects of ZPED for essential drugs on healthcare costs and utilization in China. Based on the included studies, we showed that there were considerable decreases in both the drug cost and the total expenditure per patient visit. Medical services also revealed increasing levels of inpatient visits annually. In China, the economic benefit of prescribing medicines was most regularly referred to as a reason potentially causing the illogical use of medicines in a previous systematic review (18). However, the improvement of the reasonable use of medicines still has many unpredictable deficiencies. ZPED covers national-level medicines, while lower-level governments develop the list according to local requirements. The main effect of the medical policy is to avoid hospitals not only being seldomly sufficiently reimbursed but also from having to deal with financial embarrassments due to the complicated medical circumstances involved (19).

ZPED has been proven to decrease the medical costs of patients, leading to the reform of the inpatient and outpatient structures (20). Hospitalization does not depend on the cost of outpatient care (20); however, both outpatient medical care and inpatient medical care are determined by medical factors, such as the health status of patients. Outpatient care is a short-term medical service that does not require an overnight stay in a hospital or a medical facility. Meanwhile, inpatient care involves continuous processes between patients and medical staff, in which the perception of the interaction of inpatients between the environment and service process is valued (21). Further studies should be conducted to explore the improvement of the medical care service system, the public health system, and the drug supply system. In addition, the results from the behavioral economic studies have indicated that people often make decisions according to not only absolute but also relative changes in price (5, 22). This manner is at the polytechnic of relative thinking theory, which shows that people are affected more by relative changes than absolute changes in a given initial stage (5).

In addition, the results of the selected studies concern the cost containment policy of healthcare, which changes prices for drugs and medical services. Changing prices is a broadly applied policy instrument in most Western countries (10). Rules and regulations of price alone would not yield a successful decrease in expenses because healthcare providers could avoid regulations by message merit. It is a fact that China experiences pressure regarding healthcare. Increasing the provisions of other medical services by increasing prices may compensate for the loss of revenue in most public hospitals in China (10).

### Clinical Practice of Zero-Markup Policy

This systemic review showed that there has been a major improvement in drug costs and the number of patient visits in spite of there being some heterogeneity in terms of total expenses. According to these findings, medical teams should set up a customized agreement to manage or decrease medical expenses using ZPED due to the growth or reduction in healthcare costs primarily being dependent on hospital practices (23). For example, both outpatient and inpatient payments were reduced in health centers in towns but not in those of county status or above (8). The monitoring system should include a longitudinal evaluation with advanced and alternative approaches for the improvement of expenditure.

The zero-markup policy for essential drugs plays an important role in decreasing the cost of drugs for chronic non-communicable diseases, such as type 2 diabetes, hypertension, metabolic syndrome, coronary heart disease, and cancer (21). In China, urban employees with basic medical insurance cannot afford outpatient expenses. Outpatient expenses could be deducted from the amount in the personal account, such as a partial return of the paid amount (16). A previous study also demonstrated that the abolishment of the drug markup fee at public hospitals has more predictable benefits for patients if they are urban employees with basic medical insurance (24). In addition, due to the limited reimbursement rate for patients who are urban residents with basic medical insurance, such patients may be more inclined to purchase drugs independently outside of the hospital, meaning that the execution of the ZPED in tertiary hospitals is less effective on medical costs. In contrast, urban employees with basic medical insurance may have a relatively higher awareness of reasonable drug use or may be more willing to make the decision to purchase drugs at hospitals (16).

### Methodological Considerations

Several methodological perspectives should be addressed when applying the findings of this systematic review. First, the relatively small number of selected studies limits the power of our conclusions. Second, the included studies vary in terms of methodological quality, which may have introduced some risk of bias. Third, from a statistical viewpoint, it is worth using either qualitatively or statistically presented aggregated evidence; however, it would be difficult to conduct a meta-analysis of the selected studies because the included studies do not provide consistent information. Finally, the findings might not be able to be generalized to other medical institutions, as the studies we included were conducted in only a few areas of China. The external validity of our outcomes should also be further explored.

### Conclusions

Although the government could implement ZEPD with the lower medical cost and drug cost to patients, and sustained income for health facilities, we have limited understanding of whether the increase in medical services was induced by the provider or was a response to unmet needs in the population. Further, studies using rigorous and advanced methods to study health policy, patient behaviors, provider behaviors, and government decisions are warranted.

## Data Availability Statement

The raw data supporting the conclusions of this article will be made available by the authors, without undue reservation.

## Author Contributions

W-YL, C-HH, T-JL, P-EC, T-HT, and C-WC conducted the study and drafted the manuscript. W-YL, C-HH, BZ, and T-JL participated in the design of the study and performed data synthesis. P-EC, BZ, T-HT, and C-WC conceived the study and participated in its design and coordination. All of the authors read and approved the final manuscript.

## Conflict of Interest

The authors declare that the research was conducted in the absence of any commercial or financial relationships that could be construed as a potential conflict of interest.
